# A cross-taxonomic explanatory framework for mobbing behavior

**DOI:** 10.1093/beheco/araf153

**Published:** 2025-12-22

**Authors:** Nora V Carlson, Hans Slabbekoorn

**Affiliations:** Department of Biology, Cunningham 202, 3800 Finnerty Road, University of Victoria, Victoria, BC, Canada V8P 5C2; Research Center for Advanced Science and Technology, 4-6-1 Komaba, Meguro-ku, The University of Tokyo, Tokyo 153-8904, Japan; Institute of Biology (IBL), Rapenburg 70, Leiden University, Leiden EZ 2311, The Netherlands

**Keywords:** antipredator strategy, communication network, mobbing, predator-prey interactions, environmental conditions

## Abstract

Mobbing is an important antipredator strategy wherein prey approach harass and attack nonhunting predators, using conspicuous stereotyped movements and/or vocalizations. This behavior can reduce current and future threats of predation. In this paper, we aim to provide a framework that integrates prey, predator, and environmental factors, to illuminate how multiple factors and their interactions can explain mobbing propensity. We hope to encourage targeted and systematic investigation into the ecology and evolution of mobbing by focusing on an integrated view on life history, social, and ecological conditions, and a broader taxonomic spread of investigations. By incorporating a broader view of an animal's ecology, we can better understand the tradeoff that individuals experience when deciding to engage in mobbing, and by examining this behavior across different species, life-histories, ecologies, and communities, we can better understand the larger ecological contexts in which mobbing is an effective strategy as opposed to when it is not. Finally, we highlight some other areas we feel need further investigation to advance our understanding of mobbing.

## Teaser

Mobbing occurs when prey harass predators, typically noisily and with others. The behavior is studied most in birds but found in many species from mammals to bacteria. It is currently poorly understood why only some animals mob. We propose three factors, and their interactions, critical to determining if mobbing is a likely response to a predator: prey factors (e.g. social or solitary), predator factors (e.g., specialist or generalist), and environmental factors (e.g., season).

## A general overview of mobbing, related behaviors, and its ecological importance

**Figure 1:**
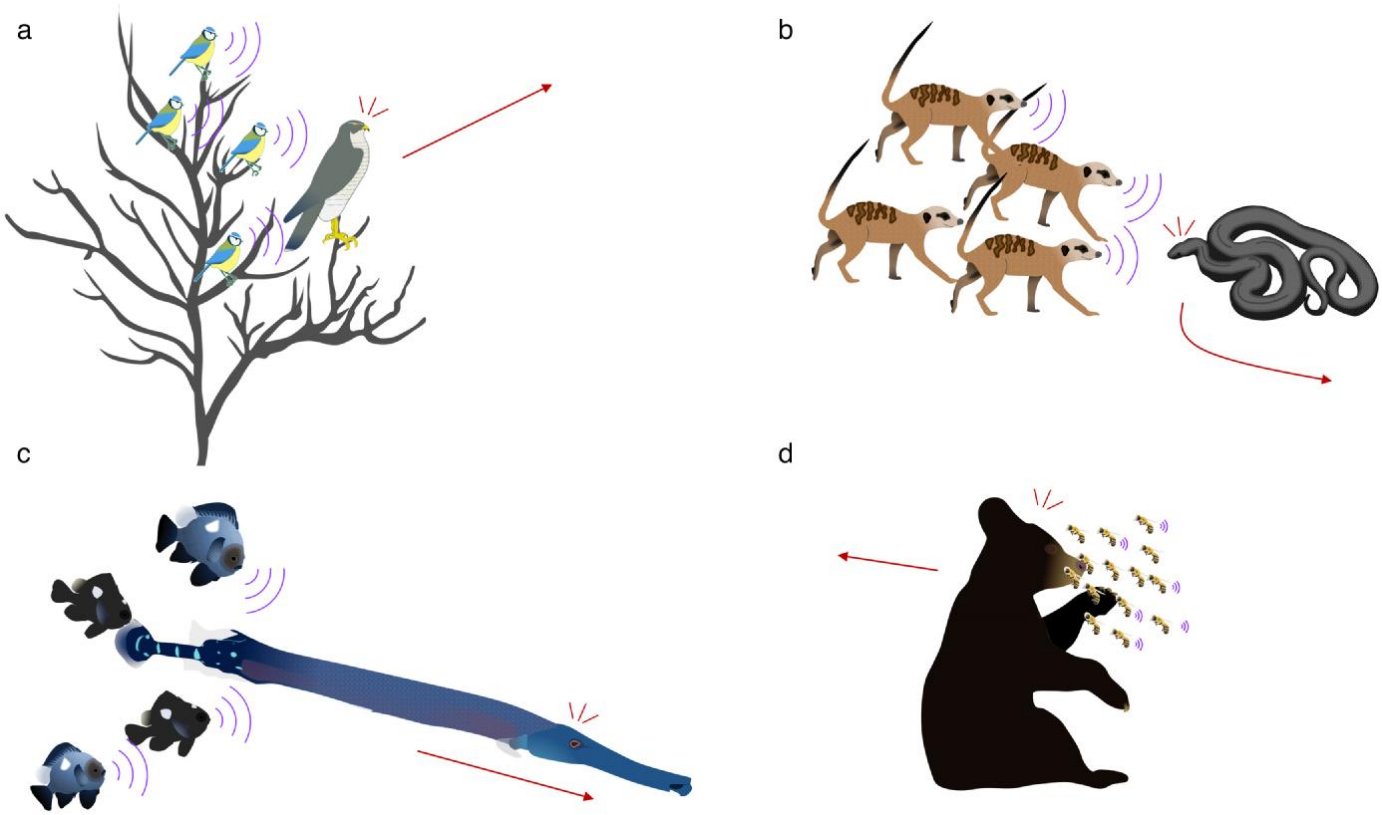
Mobbing examples across taxa. Examples of mobbing in a) birds (blue tits mobbing a sparrowhawk; Carlson et al. 2017a), b) mammals (meerkats mobbing a mole snake; Graw and Manser 2007), c) fish (three spot damselfish mobbing an Atlantic trumpetfish (Helfman 1989), and d) insects (honeybees mobbing/swarming a bear; Fuchs and Tautz 2010). Red arrows indicate the predator moving away from the mobbing group.

Mobbing is an antipredator strategy wherein preyed-upon species will harass and even attack a predator and thereby reduce the current and future threat of predation, using conspicuous stereotyped movements and/or calls (Fig. 1; Evans et al. 1993; Templeton et al. 2005; Carlson et al. 2017a; Carlson et al. 2018; Carlson and Griesser 2022). It not only is an effective antipredator strategy, mobbing also facilitates increased foraging opportunities and survival (Goodale and Ruxton 2010; Griesser 2013; Gentry et al. 2019), buffers habitat fragmentation (Sieving et al. 2004), facilitates learning about novel predators (Curio et al. 1978; Baker 2004; Szymkowiak 2021), allows collective assessment of current risks (Graw and Manser 2007; Driscoll et al. 2022) is thought to create large communication networks that are beneficial to the species community to learn about and recognize predators and avoid predation (Goodale and Ruxton 2010; Carlson et al. 2018, 2020b), and is thought to support community structure and function (Goodale et al. 2010; Carlson and Griesser 2022). Mobbing seems present in almost all animal taxa, although the literature is heavily biased to birds (∼74% of all studies), with some attention to mammals (∼19%), and very few studies on fish (∼5%), insects (∼1.5%), reptiles (∼0.5%; Carlson and Griesser 2022), and even one study on bacteria (Shteindel and Gerchman 2021). There is empirical evidence that mobbing can effectively reduce both the short- and long-term threats of predation (Pavey and Smyth 1998; Rebollo et al. 2023). Therefore mobbing is thought to have evolved primarily to lower the risk of predation to self and/or others, including kin and group mates, often by either physically removing a predator, disrupting predator hunting, or communicating with prey about the predator (or in one case about their own quality; Curio 1978; Carlson et al. 2018). Although it is effective, it is not without risk (Smith 1969; Motta-Junior 2007). While many aspects of mobbing appear the same across taxa (ie approaching and harassing or attacking a predator), some aspects vary inherent to variation in body plan (ie whether they are bipedal, can fly or swim, etc.), habitat type, sensory capacity, and communication potential. For example, many mobbing insects, fish, and reptiles may not have a vocal component to mobbing (though fish may vocalize while mobbing; Ladich and Myrberg 2006; Ladich 2019). Differences in environment (eg in air, underwater, on plants, etc.) may alter the ways in which individuals communicate about or engage with predators (eg chemical or vibrational communication). However, while mobbing is an important strategy for escaping depredation, current research in this field tends to focus on very specific aspects of the behavior (Carlson and Griesser 2022). The majority of current research in mobbing overlooks its general ecology, inter- and intraspecific variation, the costs and benefits of mobbing, and its importance across a wide range of species and ecosystems (Carlson and Griesser 2022). This lack of examination of mobbing ecology, variation, and tradeoffs leaves gaps in our ability to understand variation in mobbing or address questions of how mobbing may have evolved and what conditions maintain it so widely across the animal kingdom. In this review, we aim to create a framework that integrates three major forces for mobbing and their interactions: prey factors, predator factors, and environmental factors. Our goal with this framework is to encourage a targeted and systematic approach to the study of mobbing to allow for future research into how it evolved and is maintained across the animal kingdom, and to encourage investigation into areas in need of further study.

## Factors explaining the propensity to mob

**Figure 2:**
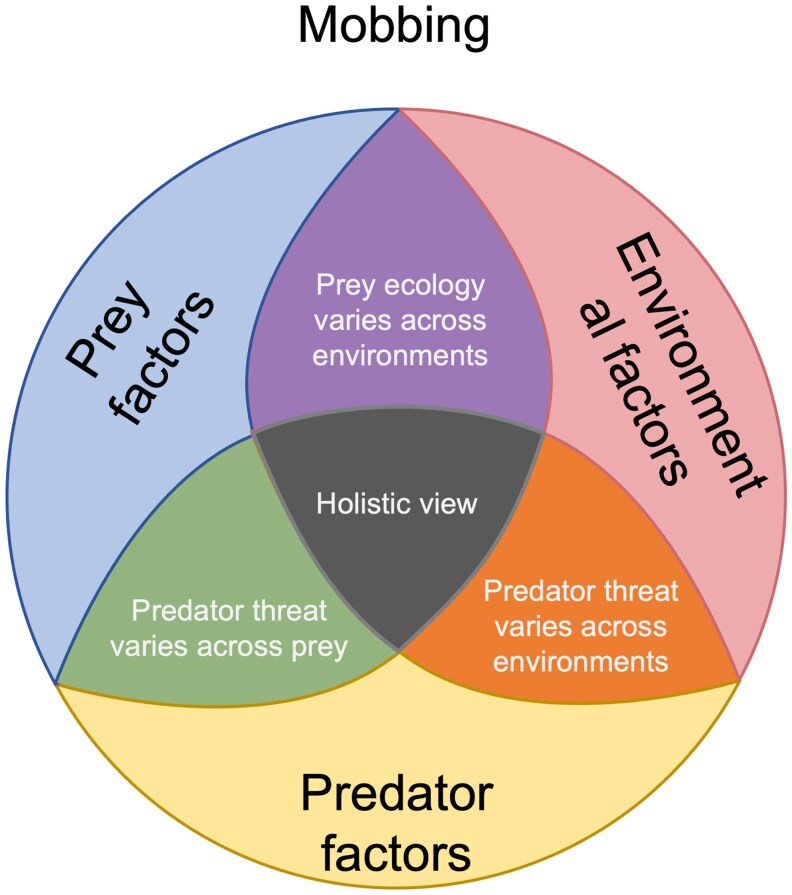
Factor categories impacting mobbing propensity. Diagram illustrating the three main factors (prey factors, predator factors, and environmental factors) and their interactions that impact the propensity of a species to engage in mobbing.

Mobbing can be explained by many factors, which can be categorized into: prey factors, predator factors, and environmental factors, and the interactions among them (Fig. 2). While many studies examining ecological similarities in mobbing among species include one or two of these categories, to our knowledge, none have addressed all three. For example, many studies have focused on prey ecology (Butler et al. 2022) but have not taken into account environmental factors like seasonality (Altmann 1956; Sandoval and Wilson 2012; de Lima et al. 2018) or predator factors (Motta-Junior and Santos-Filho 2012; Sandoval and Wilson 2012; Dutour et al. 2017; de Lima et al. 2018). To allow for a targeted systematic approach to mobbing research in order to understand the evolutionary forces that gave rise to mobbing behavior in different species, it is necessary to apply a framework to new research examining how aspects of prey and predator ecology and environmental influences all impact mobbing propensity (Fig. 2).

### Prey factors

The first set of factors to consider relate to the ecology of the mobber, such as species-specific food choice, habitat use, and predation vulnerability. In many birds, various features related to foraging ecology have been shown to predict which species in forest communities are more likely to engage in mobbing (Dutour et al. 2017; da Cunha et al. 2017b; de Lima et al. 2018; Butler et al. 2022). Species that forage in locations where they are more able to escape the predators they mob, in the case of birds—ones that spend time in the canopy or understory rather than the ground (Ekman 1986; Selås 1993; da Cunha et al. 2017b), and in primates—ones that can more easily escape into trees (Crofoot 2013), are more likely to mob. Similarly, bird species that have diets that impact their ability to detect aerial predators, limit their space use to dangerous areas (eg the ground; Ekman 1986; Selås 1993; da Cunha et al. 2017b), or are shared by many species resulting in collective/cohesive groups (eg a more varied diet or one of primarily insects, fruit or seeds; da Cunha et al. 2017b; de Lima et al. 2018; Butler et al. 2022), are more likely to mob and/or with greater intensity than those that are less vulnerable to predators due to where they spend their time or what they eat. Additionally, bird species which make up a larger portion of a predator's diet are more likely to mob (Dutour et al. 2017; da Cunha et al. 2017b; de Lima et al. 2018). Species' food choice and habitat use influence their exposure to predators which can increase their risk of predation. For example, birds in the canopy are likely to encounter a perched predator that is not actively hunting as they share the same space and/or may be more difficult for predators to ambush (Ekman 1986; da Cunha et al. 2017b). Birds that forage on the ground, however, are more vulnerable to raptor predation (Ekman 1986; Selås 1993), and likely to encounter an avian predator most often when it is hunting/attacking. If prey does not encounter a predator outside of an immediate attack, it has no chance to mob. These specific features suggest that, in general, species at high risk from a particular predator are more likely to mob that predator, as long as they have the chance to encounter that predator before being attacked by it.

**Figure 3:**
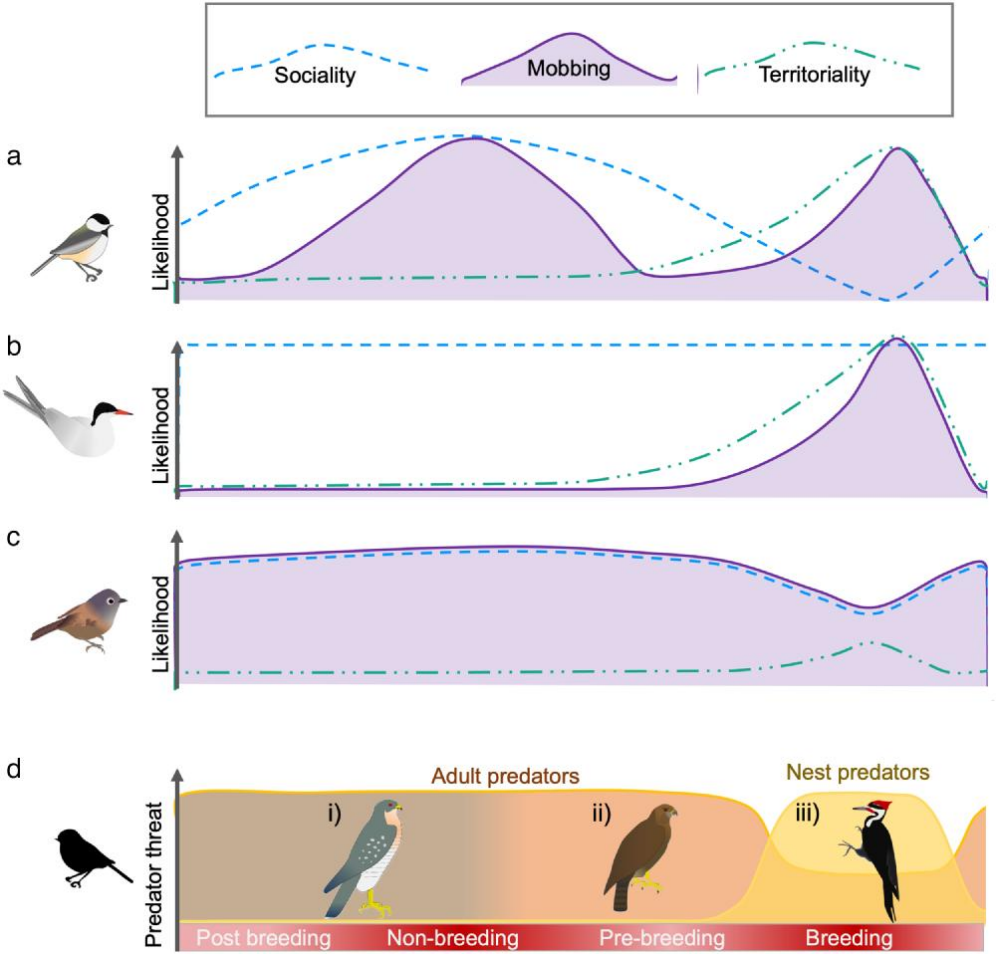
Influence of seasonality and both predator and prey ecology on propensity/likelihood to mob. Examples of the interplay between environmental factors (in this example across breeding season) and prey factors (degree of sociality - - -, and territoriality − · · −) of a species with different prey factors. Species a) has multiple peaks of mobbing propensity (−−−) across the breeding and nonbreeding season. For example, black-capped chickadees have two peaks of mobbing, one during the breeding season when territoriality is high, and one in winter when sociality is high (Shedd 1983). Species b) has one peak of mobbing propensity during the breeding season. For example, Arctic terns are gregarious throughout the year but territorial at their nests in the colony during the breeding season (Clode et al. 2000; Hatch et al. 2020). Species c) has a consistent mobbing propensity across the year with a slight decrease during the breeding season. For example, David's fulvetta are a main member of stable year-round mixed-species flocks throughout the year and often initiate mobbing in the group. However, as they are somewhat more restricted in their movements as they need to feed young during the breeding season, they tend to slightly decrease the time spent in these flocks and, therefore participating in mobs, during the breeding season (Jiang et al. 2020). Finally, d) provides an example of the interplay between seasonality and predators factors, illustrating the different threat (−−−) predators may pose across the season, due to both changes in predator diet as well as movement of predators and prey. During the nonbreeding season, adult predators di) and dii) pose a high risk. However, due to migration of predators and/or prey, the predators a prey species encounters may change across the season from predator di) to predator dii). The breeding season also affects which species pose a risk. Adult predators di) and dii) may pose less of a risk in the spring if they shift preferences to young or other taxonomic groups such as insects, while nest predators diii) become a risk to young impacting adult fitness even though they pose no direct risk to adults.

**Figure 4:**
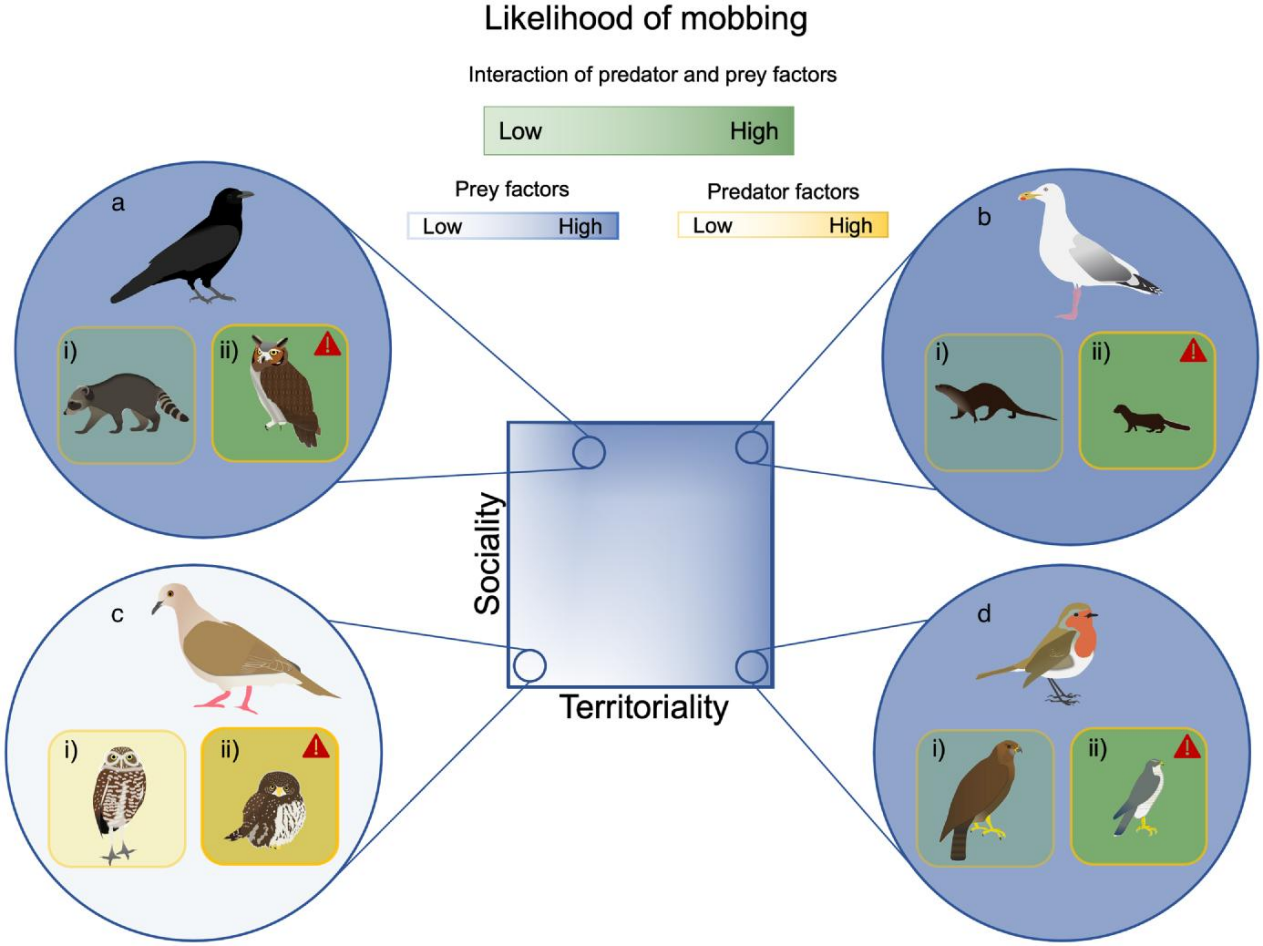
Influence of predator and prey factors on a species' propensity to mob a particular predator illustrating that there are multiple interactions of prey and predator factors that will lead to high probability to mob. Figure shows an increase (color saturation) in the likelihood of mobbing as result of the interplay between prey factors (territoriality and sociality) and predator factors (danger) of different species. Species a) is highly social but not very territorial. For example American crows during the nonbreeding season encountering a i) low-threat predator such as a racoon and a ii) high-threat predator such as a great-horned owl which can pose a threat year-round (Yorzinski and Vehrencamp 2009). Species b) is a highly social and highly territorial species. For example, gulls during the breeding season encountering a i) low-threat predator such as an otter and a ii) high-threat predator such as an American mink dangerous to both adults and young (Clode et al. 2000). Species c) is a nonsocial nonterritorial species such as a white-tipped dove encountering a i) low-threat predator such as a burrowing owl which poses no threat and a ii) medium-threat ferruginous pygmy-owl which can depredate many species (da Cunha et al. 2017b). Species d) is a nonsocial and highly territorial species. For example, a European robin, (Lack 1948) encountering a i) low-threat predator such as a buzzard, which poses little threat and a ii) high-threat predator such as a sparrowhawk (Carlson et al. 2020b) which is a small bird specialist.

Social tolerance and interactions is also likely key in modulating a species' propensity to mob (da Cunha et al. 2017b). While some studies have shown that an audience, both conspecific and heterospecific, can impact mobbing propensity and intensity (da Cunha et al. 2017a; Carlson and Griesser 2022), how this is tested and defined varies widely, often causing confusion about how sociality impacts mobbing behavior. For example, in one study, a factor based on a species' specific conspecific social system did not predict whether it mobbed while a factor based on a more general measure of sociality did (da Cunha et al. 2017b). To better tease out how inter- and intraspecific interactions affect the costs and benefits of mobbing and therefore species mobbing propensity, we propose examining this through the broader lens of sociality and territoriality (Figs. 3 and 4). Sociality, by its very definition, necessarily includes others, often conspecifics, but heterospecifics as well (Ward and Webster 2016; Goodale et al. 2017). Territoriality, on the other hand, necessarily excludes others, often conspecifics, but heterospecifics as well (eg Reif et al. 2015; Sells and Mitchell 2020; Maher 2022). However, as both sociality and territoriality can be measured across conspecifics and heterospecifics, and across spatial and temporal scales, what emerges is a graded interaction of territoriality and sociality for different species and contexts (Figs. 3 and 4). We can see how this works by examining the costs and benefits to both territoriality and sociality.

If a species is territorial, in that they are more closely tied to a location (ie defending a resource; Maher 2022) the cost of abandoning that area is usually high (often resulting in decreased fitness; Maher 2022). As many territorial species would suffer large costs of abandoning a territory in the face of predation (Owen-Smith 1977; Ostfeld 1990; Maher 2022), abandoning an area in response to predator presence is unfeasible and removing a predator is, therefore, highly beneficial. In fact, predation is rarely discussed in theories of territory establishment and maintenance, suggesting it is often unimportant in decisions regarding territory abandonment (Owen-Smith 1977; Ostfeld 1990; Maher 2022). On the other hand, if a species is not territorial at all (ie, is not restricted to a particular location due to resources), then they can leave the area to avoid a predator without incurring the same costs. Nonterritorial male water skinks, *Eulamprus heatwolei*, for example, are more likely to flee simulated predator attacks than territorial ones (Stapley and Keogh 2004). However, while the benefits of removing a predator may be higher, the costs are also likely higher, as without a group to assist in mobbing, the risk for a territorial mobber is greater as they lack the dilution and confusion effects of the group when encountering a predator (Hamilton 1971; Curio et al. 1978; Caro 2005; Crofoot 2013; Carlson and Griesser 2022).

If a species is social, the cost to mobbing is lower as species living in groups may be more likely to receive reliable help during a mobbing event (eg, individuals are more likely to produce mobbing calls with familiar individuals; Krams and Krama 2002; Coppinger et al. 2018), thereby reducing the risk of being attacked by the predator through dilution and confusion effects (Hamilton 1971; Foster and Treherne 1981). Individuals in large groups often have large territories or more resources to be able to meet group demand, allowing for farther escape from a predator without abandoning a territory. However, with more individuals competition over resources typically also increases, thereby increasing the cost of abandoning a territory, and increasing the benefits of driving away a predator. Larger groups are often also more conspicuous, and so predator encounters in groups may therefore increase, increasing the benefits of long-term changes in predator behavior as a result of mobbing (Ekman 1986; Pavey and Smyth 1998). Many group-living individuals also gain indirect benefits of removing predators by protecting group-mates who are often kin or breeding partners (Griesser and Ekman 2005; Griesser 2013; Griesser and Suzuki 2017).

These different costs and benefits of territoriality and sociality may directly affect the tradeoff between the costs and benefits of mobbing for any species (Curio 1978; Crofoot 2013; Carlson and Griesser 2022). Examples of territoriality and sociality interacting can be seen in a range of species. For example, many species establish individual territories which are aggressively defended against conspecific outsiders (often except for one other individual or offspring, thereby exhibiting some sociality; Clutton-Brock et al. 1988; Foote et al. 2004; Maher 2022). These territories are sometimes restricted only to a defended den/nest, other times they include food and other resources as well (eg nests in many flocking birds, lekks in some ungulate species; Clutton-Brock et al. 1988; Foote et al. 2004; Maher 2022). In many species considered highly social, like social primates and cooperatively breeding species, group-mates are highly social, but groups themselves express high levels of territoriality against neighboring groups (Dow 1977; Arnold 2000).

Who sociality occurs with, heterospecifics or conspecifics, likely also play a role in the propensity to mob. Some species are universally aggressive, such as Anna's hummingbirds, *Calypte anna*, which attack anything entering their territory (Ewald and Carpenter 1978; Clark and Russell 2020). Other species may be conspecifically territorial and heterospecificly social like European robins, *Erithacus rubecula* (Lack 1948) and European rollers, *Coracias garrulus* (Gameiro et al. 2022), who will temporarily associate with mixed-species groups, but aggressively defend territories against conspecifics. Species may also be conspecifically tolerant and heterospecificly intolerant such as noisy miners, *Manorina melanocephala*, which live in large conspecific flocks but aggressively remove heterospecifics from their territories (Dow 1977; Arnold 2000). Each of these combinations of territoriality and sociality must be considered, as simply defining sociality in specific limited contexts such as family structure or group size may overlook a species' functional sociality. A conspecificly solitarily and territorial species that joins with a heterospecific group within its territory may experience similar benefits of sociality as does a conspecificly social species that is not highly territorial and does not associate with heterospecifics. In this case, the benefits and costs to mobbing may be very similar between the two species even if the two species are classified differently when discussing specific categorizations of sociality and territoriality.

These different costs and benefits of territoriality and sociality in the context of mobbing and antipredator behavior in general, likely directly affect the propensity for a species to mob (Curio 1978; Crofoot 2013; Carlson and Griesser 2022). In fact, although not explicitly tested, mobbing is thought to be more common, and is therefore more commonly studies in both territorial (eg very common during the breeding season in birds when they are more territorial; Verea 2023) and social/gregarious species that occur in larger groups (da Cunha et al. 2017b; Ippi et al. 2022; Hernndez et al. 2024) or species with other close associations with group mates (Alatalo and Helle 1990; Butler et al. 2022). However, as no known comparative studies focus on how territoriality and sociality may interact across species and contexts have ever been done, this remains untested.

### Predator factors

**Figure 5:**
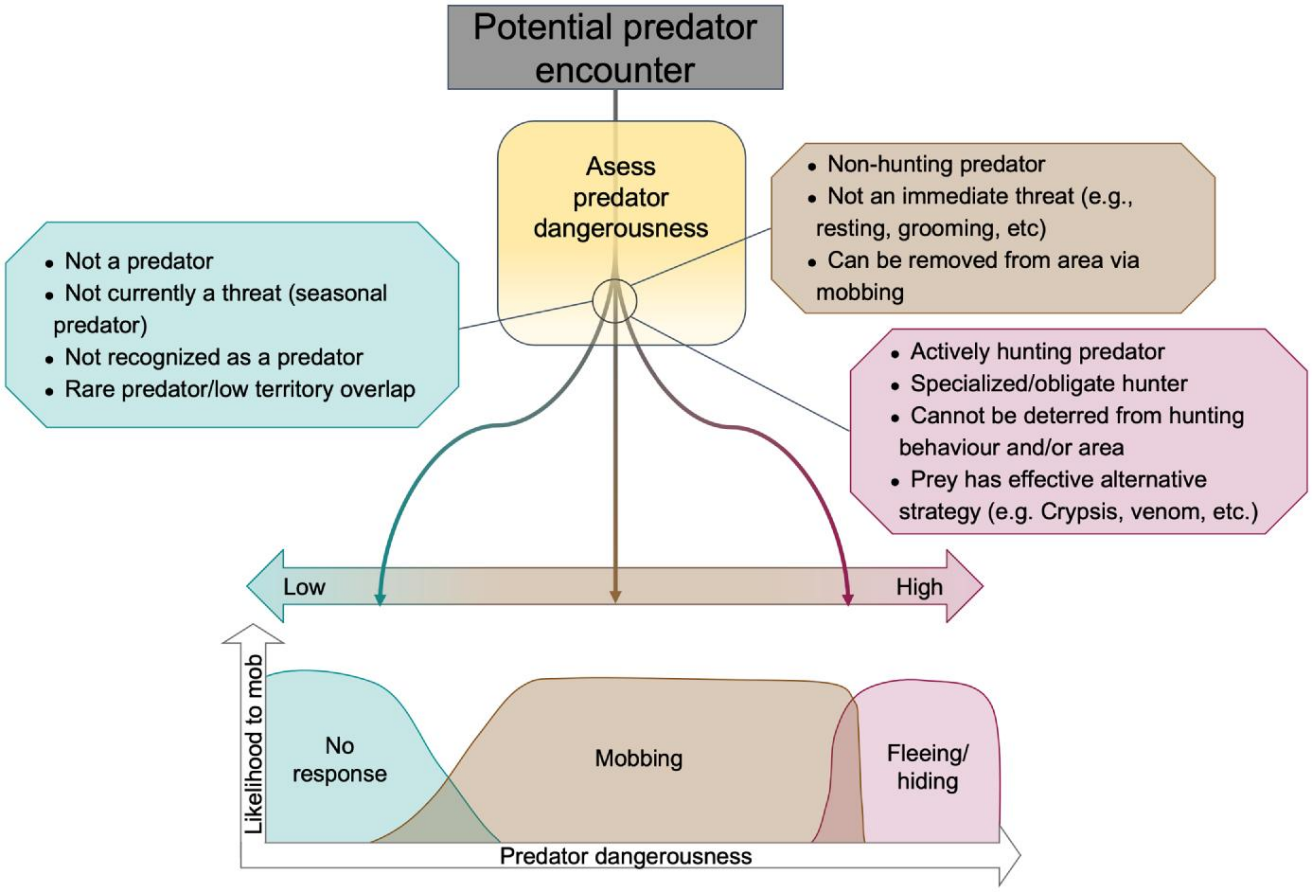
Factors determining the response to a potential predator encounter. Figure illustrating the factors determining whether no response, mobbing, or fleeing/hiding is the most effective strategy when encountering a potential predator and assessing their dangerousness.

The second set of factors relate to variation in the type and state of the predator (Lima 2002), which is arguably one of the most important and least well-studied factors. Predator foraging strategy and diet specialization affect the danger to their prey species, and thereby the costs and benefits of mobbing (Caro 2005; Dutour et al. 2017; de Lima et al. 2018). This is so important because there are many ways in which mobbing may not be a beneficial antipredator strategy (Fig. 5). The immediacy of danger can make mobbing too risky and often results in fleeing (Griesser 2008). For example, if an actively hunting predator is encountered, many species will hide or flee, rather than draw attention to themselves (Marler 1955; Caro 2005). Mobbing is also ineffective if the predator remains as dangerous when detected and mobbed as when un-detected and left undisturbed. If mobbing by prey would not affect a predator's effectiveness then it becomes a useless strategy. Mobbing is thought to be most effective when there is a chance of survival. For example, if a predator is encountered in a context where it is less dangerous, such as while sleeping or grooming, or if mobbing will affect a predator's effectiveness when actually hunting prey (Marler 1955; Klump and Shalter 1984; Caro 2005) then it becomes a useful strategy.

Predator foraging strategy is an important factor to consider when considering a species' propensity to mob as some foraging strategies negate any benefits of mobbing. Mobbing is thought to be most effective on ambush predators and other predators that rely on prey not noticing them before they attack, as their chance of success is reduced when prey notice and draw attention to (mob) them. However, for strategies where prey discovering a predator does not dramatically impact its success, mobbing will remain ineffective, and potentially even increase predator success when prey approach the predator. This is often the case with predators that are specialists of a particular species.

Diet specialization is also important to consider as not all predators represent a similar threat. Predation pressure and the likelihood of encountering a predator may directly impact the benefits of mobbing through its effect on changing future predator behavior. Predators that specialize on species A, for example, pose a larger threat to species A than to another species B. Due to such a predation bias, species A would benefit more from their predators leaving the area than species B. This means that species A would be more likely to mob the predator than species B (Caro 2005; Dutour et al. 2017; de Lima et al. 2018). Similarly, rare predator species pose less of a consistent and easily recognized threat to the local prey community and are therefore less likely to be mobbed (Sandoval and Wilson 2012; Tilgar and Moks 2015; Carlson et al. 2017b). If a predator is only encountered rarely, expending the energy cost to drive it away and alter its future behavior may not outweigh the cost of simply avoiding it during a rare predation event.

Predator density may also affect mobbing decisions. If removing a predator within an individual or group's territory/home range does not lower the overall predator density, then mobbing is not an effective strategy. For example, if a predator that is chased away is immediately replaced by neighboring individual or attracts a new predator (Smith 1969), then mobbing has not effectively lowered current and future predation pressure.

### Environmental factors

The third set of factors to consider relate to habitat type and seasonality. Environmental factors play an important role in most predator-prey interactions, as predator and prey presence and behavior may vary across habitats and/or seasons (ie, yearly changes in vegetation, temperature, resource availability, etc.). However, environmental factors are often overlooked when evaluating mobbing propensity (though see Shedd 1982; Shedd 1983; Zimmermann and Curio 1988; Ferrari and Ferrari 1990; Dutour et al. 2019; Jiang et al. 2020).

Habitat type can affect species vulnerability though the presence of refuges and mutual visibility for predator and prey (Ekman 1987), therefore directly impacting the risks of mobbing. Mobbing species are sensitive to habitat structure when mobbing (eg loss of canopy cover can decrease mobbing propensity, possibly a result of increased perceived predation risk; Hua and Sieving 2016). Refuge opportunity and visibility vary drastically between habitats, such as dense forests compared with open grassland, or between spring and winter in deciduous forests. Available cover in deciduous forests decreases during fall and winter as leaves fall which is likely to increase both predator and prey detectability. This increase in detectability could increase benefits of mobbing and lower risks of drawing attention to a group, as groups will already be more conspicuous.

Season can also play a large role in mobbing propensity, as seasons drive food availability in many species, which could alter the willingness of individuals to mob. If food resources are abundant, and individuals can move on from a particularly good food patch with little risk or cost, then mobbing becomes less beneficial as the cost of confronting a predator is not outweighed by the benefit of staying in that area. Similarly, increased food resources may also decrease the need for social tolerance in the form of mixed-species groups, resulting in smaller groups (Kubota and Makamura 2000) and less mobbing.

Seasonal changes can also affect availability of other resources, such as water in dry or water-restricted habitats, resulting in changes in habitat use by both predators and prey. In some cases, this can result in changes such as increasing grouping propensity for some species (Kubota and Makamura 2000), lowering the costs of mobbing, making mobbing more likely to occur. In other cases, more restricted resources such as waterholes in arid habitats, could increase predator-prey encounter rate while simultaneously increasing costs for fleeing from the resource and lowering benefits of mobbing as predators are unlikely to leave. This could result in overall lower propensities to mob, even in the presence of a predator, as mobbing becomes situationally ineffective.

### Prey and predator interactions

The importance of the interplay between prey and predator factors is reflected in predator dangerousness relying heavily on both the territoriality and sociality of the prey and the ecology of the predator (Fig. 4). When individuals are more restricted to a territory, they may suffer increased costs from mobbing dependent on the degree of territory overlap. If there is large or complete overlap between the territory of the prey and the predator in such a way that most space both predator and prey use is almost entirely the same, then mobbing cannot remove the predator from the area and is therefore less effective and less likely (Forsman and Mönkkönen 2001). If the overlap between areas used by predator and prey is a very small or negligible, then encounters between predators and prey will be rare and avoidance a more efficient strategy (Sandoval and Wilson 2012; Tilgar and Moks 2015; Carlson et al. 2017b). While the first case is largely untested, there is evidence that supports the second. Studies have shown that in prey populations with relatively low predator density, and thus rare predator encounters, prey species mob rare predators less often and less aggressively than in populations with relatively high predator density (Tilgar and Moks 2015; Shteindel and Gerchman 2021). In this way, territory overlap of predators and prey can modulate mobbing propensity, even when other predator factors such as hunting strategy would predict a different outcome.

As with territoriality, increased sociality (conspecific or heterospecific) can make some of the potential benefits of mobbing increase and some of the costs decrease (Fig. 4). Although larger groups are more conspicuous, possibly attracting greater attention from predators (Ioannou and Krause 2008), they also decrease the risk to individuals by both increasing predator detection and diluting individual risk (Caro 2005; Goodale and Ruxton 2010). Additionally, larger groups are known to be more effective at driving away predators. For example, hyenas are successful at mobbing lions when they outnumber the lions four to one but generally do not even try mobbing if there are < two to one (Cooper 1991). Large group size therefore could raise the general propensity of mobbing, independent of a predator's hunting strategy, dangerousness, and diet, and could result in cases of mobbing by large groups when it would be considered a less effective strategy for small groups.

The life history, hunting strategy, and morphology of a predator all impact the type of threat or risk posed to a particular prey (often based on that prey's life history, antipredator strategies, and morphology). This means that different predators will require different antipredator strategies (eg fleeing vs mobbing). This is evidenced in the responses of young to mobbing calls, which often have different vulnerabilities to predators, and whose responses often differ from their parents. Young will often hang back and watch while adults mob (Buitron 1983; Ficken et al. 1987; Fichtel 2008; Sternalski and Bretagnolle 2010; Griesser 2013), or even have different avoidance behaviors to different predators (Suzuki 2011). In other contexts, for example, heterospecifics that share some, but not all of the same predators will often modulate their response to one another's mobbing calls based on likely predator identity (eg black-casqued hornbills, *Ceratogymna atrata*, and Diana monkeys, *Cercopithecus diana*; Rainey et al. 2004). Additionally, predators themselves may come from different areas, needing different types of approaches—something seen in the difference in response to perched predators vs. aerial predators in birds. Perched predators often elicit a mobbing response while flying predators often eliciting a flee response (Marler 1955; Caro 2005; Carlson and Griesser 2022). These types of predator differences result in differing levels of threat posed by predators requiring different approaches by their prey resulting in variation in propensity to mob based on the “type match” of predator and prey.

Different predators may also differ in their vulnerability to mobbing, which can directly impact the effectiveness of mobbing and therefore the benefits to mobbers. For example, prey may target vulnerable areas on a predator to increase the chance of injuring them. In other cases, predators may be more robust to damage from prey. Crabs, for example, are much less likely to avoid mobbing by shrimp and instead are more neutral or aggressive (Carlson et al. 2024, unpublished dataset). Fish, on the other hand, are more likely to flinch and move away from mobbing by shrimp. Shrimp, in turn, are less likely to mob crabs than fish, which is thought to be a result of the relative danger and ineffectiveness of approaching and mobbing crabs (Carlson et al. 2024, unpublished dataset). Predators that are vulnerable to mobbing are likely more easily deterred from hunting and therefore mobbing may be a more effective strategy increasing the propensity of mobbing in prey species. However, if a predator is less vulnerable to mobbing and/or responds with increased aggression and is not deterred from hunting, then mobbing is an ineffective strategy and so prey species may be less likely to mob that predator.

### Prey and environmental interactions

Season can also play a large role in species mobbing propensity, as many species have seasonally-driven behavioral patterns, such as migration, breeding, and hibernation, which may have a strong effect on their propensity to mob (Fig. 3). The importance of the interplay between prey factors and environmental factors is best highlighted by breeding ecology, as it is one of the most obvious seasonal shifts in territoriality and sociality with remarkable taxonomic variation (Fig. 3a–c). For some species like the black-capped chickadee, *Poecile atricapilla* (Shedd 1983; Bruintjes and Taborsky 2008), two different peaks of high likelihood of mobbing occur. One in the winter as threats to self-increase and one in spring when threats to young increase. These also co-occur with increased sociality in the winter as they participate in large mixed-species flocks and increased territoriality in the spring when they defend territories (Fig. 3a). For other species like the arctic tern, *Sterna paradisaea* (Clode et al. 2000), mobbing coincides with an increase of territoriality during the spring when pairs have a nest in the colony, while sociality stays more stable throughout the season (Fig. 3b). There also are species that have more subtle shifts in territoriality and sociality (Jiang et al. 2020), with accordingly little variation in the propensity of mobbing across seasons (Fig. 3c). David's fluvettas, *Alcippe davidi*, for example, only have a small decrease in total mobbing propensity during the breeding season, which may be due to some fluvettas splitting off from mixed-species flocks to remain closer to their nests, while others remain with the mixed-species flock (Jiang et al. 2020). Socially living species like many primate and mongoose species known to mob (Graw and Manser 2007; Crofoot 2013), may also be less affected by seasonality effects of territoriality/sociality if individuals do not alter their social relationships with seasonal events such as being pregnant (Tamura 1989; Swaisgood et al. 1999) or having young (Phillips and Waterman 2014). These species-specific interplays of territoriality and sociality with seasonal fluctuations in mobbing conditions can be thought of in terms of broad categories of breeding ecology. Colonial breeding is defined by individuals being territorial around the nest but highly social within the colony (Clode et al. 2000; Guiden et al. 2019). Cooperative breeding is defined by individuals being highly social near the nest, but hold a larger territory which they defend against other conspecific groups (eg *Neolamprologus pulcher,* Bruintjes and Taborsky 2008; Cant 2012). Pair breeding is defined by pairs of individuals (somewhat social), which defend a shared territory against conspecifics (Shedd 1983). Finally, antisocial breeding and lekking species are territorial against all other individuals at their territory or lek, sometimes including breeding partners, when not actively courting/copulating (eg Anna's hummingbird; Ewald and Carpenter 1978; Clark and Russell 2020), though in some species, fallow deer for example, females remain somewhat social off the lek (Imperio et al. 2020). In all of these cases, one may expect an increase in mobbing, as either increased proximity to others that may assist in mobbing, or increased territoriality may increase benefits of mobbing and make it an attractive strategy. However, each strategy varies in their costs and benefits and thereby in the propensity of mobbing. For example, many colonial nesters have a mobbing approach where only those pairs closest to the threat, or that have young, engage in any mobbing behavior as a predator moves through the colony (Dominey 1983; Shields 1984). Other individuals will come from farther away to observe, learn and, sometimes, assist a bit, but will often leave the mobbing to those most in danger (Shields 1984; Clode et al. 2000). In pair breeders, the pair are the primary mobbers when a predator appears, sometimes with known neighbors (conspecifics or heterospecifics) providing some assistance, (Zimmermann and Curio 1988; Krams et al. 2006; Jiang et al. 2020).

Seasonal changes to habitat use and behavior as individuals shift priorities to prepare for breeding or overwintering can also affect the costs and benefits of mobbing. For example, singing and other conspicuous visual displays that are part of courtship for many species (Ekman 1986; Campos et al. 2009) occur in highly visible areas including tops of trees and open ground, making the signaler more vulnerable. During this time, many species also increase their territoriality, often driving conspecific neighbors out of their territory, thereby increasing their overall risk when mobbing as they may have less help. Species communities can change during this time as well as many individuals either migrate to a new area with different species, or different species arrive at resident individual's area as immigrants. Frequently this will result in different species available to assist in mobbing efforts. In some areas, however, it can potentially reduce overall mobbing helpers as many species only respond to the mobbing calls of familiar species (Nocera et al. 2008). This coupled with potentially unknown predators, could increase risk and decrease benefits of mobbing when in more unfamiliar territory, resulting in lowered mobbing propensity.

### Predator and environmental interactions

The importance of the interplay between predator factors and environmental factors is reflected best in the effect of seasonality on predator-prey dynamics, through the fluctuating threats posed by migratory predators and shifts in predator diet (Fig. 3d). The predators that pose a threat to prey species change as predators move across different landscapes, thereby changing spatial overlap and encounter rates with prey (Fig. 3d i, ii). Some predators are present year-round, others just in summer or winter, while even others are only encountered when prey species are migrating themselves (Fig. 3d i, ii). Predator diets also often shift, sometimes from one taxonomic group to another such as from small mammals to birds (Masoero et al. 2020). Other predators may shift the demography of their prey, catching more young than adults as young are often less able to escape (Sullivan 1989). These changes alter the costs and benefits of mobbing for adult prey, changing which predators they are likely to mob, and how intensely (personal observations). For example, if a predator poses little threat to an adult bird in the winter as it primarily forages on small mammals, but a large threat in the spring when it switches to primarily eating small birds, avian prey may also decrease and increase their propensity to mob that predator in line with the threat it currently poses.

Finally, for breeding prey, the predator landscape alters dramatically due to both the seasonal risk of nest predation by species that otherwise pose no threat to adults and predators that change their preference to young when they become available. Nest predators post a high risk to reproductive success, but not to adult survival. This would suggest that the benefits of mobbing should increase, while the risks decrease, thereby increasing the change of mobbing. And while there do appear to be many species that mob only when young are involved, not all species do this. One reason could be different costs to mobbing in the breeding season, specifically the cost of attracting more predators to cryptic nests (Krams et al. 2007). Furthermore, many nest predators still pose a danger to adult prey as they can and will kill them. Therefore, while the benefits to removing predators can increase, costs of mobbing may stay largely the same, or also increase if mobbing attracts other predators or nest predators also kill adults (Fig. 3b iii).

## Where do we go from here?

This review is an attempt to create an integrated framework of three ecological factors that may affect the propensity of mobbing and aims to encourage a targeted and systematic approach toward investigating how mobbing evolved across species. We believe that it is important to gain a better general understanding of the phenomenon of mobbing as it is so widely prevalent and important to community structure and function. To accomplish this, we suggest the following:

1. *Data collection, analyses, and conclusions*. Many different factors influence the propensity of a species to engage in mobbing. While some studies have examined specific aspects of predator and prey life history as well as seasonality, rarely are all three examined together or analysed in a systematic way allowing separation of the influences of each. Additionally, factors that are examined are often overly specific (eg breeding strategy) thereby not allowing inference for underlying mechanisms with broader application (eg social tolerance). Therefore, when investigating drivers of mobbing in different species, it is imperative to keep in mind predator, prey and environmental factors and their interactions, both when designing experiments and when drawing conclusions from results. Many current studies classify different species into mobbing and nonmobbing while only taking some of these factors into account, possibly overlooking predator or environmental factors that may limit mobbing expression.
2. *Taxonomic bias and mobbing*. As much of what we know about mobbing comes from studies of birds and mammals, much of what we know and hypothesize about why, when, and how mobbing occurs is colored by taxonomic bias. To better understand mobbing behavior we need to investigate mobbing across a wide variety of taxa, in as many different habitats, seasons, and predator-prey communities as possible. As this is difficult to do, research efforts could start by focusing on families of different taxonomic groups known for mobbing in different ecosystem types such as tits (Paridae) and gulls (Laridae), squirrels (Sciuridae), mongoose (Herpestidae), damselfish (Pomacentridae), and squirrelfish (Holocentrinae). By targeting widely occurring known mobbing species we can begin to compare across different ecological factors as well as to investigate how taxonomic differences in body plans may impact mobbing.
3. *Predators*. While not explicitly discussed in the above sections, understanding the effects of mobbing on predators, and how predator features impact mobbing behavior in their prey is important. We know very little about how mobbing affects predators as the majority of research has focused on prey decisions. However, by understanding predator responses to mobbing, we can better understand the effectiveness and risks of mobbing for prey.

Using this way of thinking about the variation in costs and benefits of mobbing as predator, prey, and ecological factors intersect, we can ask more targeted questions to better unveil why mobbing is such a ubiquitous and effective antipredator strategy. Moving forward we argue that we should also pay special attention to inter- and intraspecific mobbing variation as well as the importance of mobbing in urbanized and industrialized areas. By focusing on inter- and intraspecific variation, we can better understand fine-scale differences in individual decisions about mobbing, while examining mobbing in the Anthropocene can reveal how anthropogenic change may be altering costs and benefits to mobbing and impacting its prevalence in a changing world. For example, by comparing mobbing across an urban gradient or across a temporal change in anthropogenic disturbance, researchers can take an experimental approach to ecological questions of human impacts.

## Finescale variation in mobbing

While many aspects of mobbing appear the same across taxa, such as approaching and harassing or attacking a predator, some aspects of mobbing vary in response to differences in body plan, habitat type, sensory capacity, and communication potential. For example, many mobbing insects, fish, and reptiles may not have a vocal component to mobbing; though it appears that fish may be quite vocal in antipredator contexts (Ladich and Myrberg 2006; Ladich 2019; Banse et al. 2024). Differences in environment (eg in air, underwater, on plants, etc.) alters the ways in which individuals communicate about or engage with predators (eg chemical or vibrational communication). For example, sound travels very differently in air, water, and through solids (eg a leaf, the ground) which can change the distance at which individuals could detect or be recruited to a mobbing event. This could change the scale over which knowledge about and participation in mobbing can occur. Similarly, body plan will directly impact how individuals can engage with a predator. Fish often bite predators or engage in visual lateral displays (Donaldson 1984; Lachat and Haag-Wackernagel 2016; Banse et al. 2024). Mammals often engage in piloerectin and tail raising (Berger 1979; Owings and Owings 1979; Graw and Manser 2007), and may use objects in their environment to assist in attacking predators (Owings and Coss 1977; Crofoot 2013). Birds, will often engage in specific postures and movements (Hinde 1954; Altmann 1956; Carlson et al. 2020a), along with diving at predators from above (Kruuk 1964).

Mobbing is often done in a social context, and local community structure may play a large role in whether individuals engage in mobbing. While group size is known to influence costs and benefits of mobbing, many species may make additional decisions about engaging in mobbing based on who is in the group. For example, in many bird flocks, eavesdroppers will respond more to some species than others. In Sri Lankan mixed-species flocks, species tend to be equally attracted to calls of racket-tailed drongos (*Dicrurus paradiseus* Linnaeus) and orange-billed babblers (*Turdoides rufescenes*; Goodale and Kotagama 2005) but respond more quickly to drongo calls (Goodale and Kotagama 2005). Similarly, in mixed-species flocks, individual birds may invest more in mobbing depending on the mobbing instigator (Randler and Vollmer 2013). This can occur in other contexts as well. European rollers (*Coracias garrulus*) that nest in heterospecific colonies return to their nest earlier and attack predators less then solitary nesting ones (Gameiro et al. 2022). This suggests that the identity of heterospecifics and the local species community itself, may impact a species' willingness to mob.

Finally, individuals often vary in mobbing behavior. While important, individual variation in mobbing behavior has received little attention as it is often difficult to study. There are some specific demographic differences that do often impact individual mobbing propensity, including breeding status and sex. For example, among scuirrids pregnant females or females with young are more likely to mob (Tamura 1989; Swaisgood et al. 1999). In some birds and primates, males are more likely to mob than females; especially in the presence of females (da Cunha et al. 2017a; Lawson et al. 2021) or after being specifically recruited by females (Mehon and Stephan 2021), while in other species, such as pronghorn (*Antilocapra americana*), females are more likely to initiate mobbing (Lipetz and Bekoff 1980). Outside of these types of demographics, factors such as boldness can also increase an individual's propensity to mob. More explorative and bolder great tits (*Parus major*), for example, are more likely to mob a nest predator than their more timid and shy counterparts (Vrublevska et al. 2015). Social context and relationship to the signaler can also influence whether an individual mobs. Dwarf mongoose (*Helogale parvula*), for example, are more likely to mob when the recruiter is an individual with whom they are more strongly bonded (Kern and Radford 2016). Additionally, in some cases even phenotype can predict mobbing behavior (Sternalski and Bretagnolle 2010). For example, brown male marsh harriers (*Circus aeruginosus*) were more likely to serve as recruiters to mobbing events, while gray males rarely, if ever, recruited or participated in mobbing (Sternalski and Bretagnolle 2010). Understanding the costs and benefits and how they impact individual decision making could provide more context for understanding the costs and benefits that impact mobbing propensity on the species scale.

## Anthropocene and human-induced environmental change

We are currently living in the Anthropocene and facing some of the fastest and most drastic changes to the natural world in recent history due to anthropogenic environmental change (Hamilton et al. 2015; Whitehouse 2015; Seddon et al. 2016; Tucker et al. 2018; Duarte et al. 2021). Environmental change due to human activities can have substantial impact on the occurrence of mobbing, as changes in habitat, season, or communities will alter the potential for collaboration and communication, the seasonal environment, and the predator landscape. Many of these changes are unprecedented and intertwined, such as increased ambient noise levels co-occurring with habitat fragmentation (Guiden et al. 2019). Because many anthropogenic induced changes are detrimental to individual welfare or population health understanding the effects they have on mobbing in species communities may provide insights on how anthropogenic changes affect species communities directly and indirectly by altering interspecific relationships (cooperation, competition, etc.), communication (antipredator and otherwise), and decision making in other contexts as well.

First, anthropogenic noise may affect mobbing propensity and mobbing group size. In terrestrial, and possibly aquatic (Banse et al. 2024), environments mobbing events are thought to be coordinated vocally, via communication of predator threat through alarm calls (Templeton et al. 2005; Gill and Bierema 2013; Carlson et al. 2017a) and vocal recruitment of allies through mobbing calls (Gunn et al. 2000; Goodale and Kotagama 2006; Suzuki 2012a). Recent studies have indeed shown that potential allies do not respond appropriately (or at all) to mobbing playbacks when accompanied by elevated noise levels (Templeton et al. 2016; Damsky and Gall 2017; Jung et al. 2020; Tilgar et al. 2022), and mobbing attendance decreased with increasing noise in urban areas (Ewing et al. 2025) . This interference with communication, through masking or distraction, decreases the direct benefits to the signalers by lowering recruitment resulting in a smaller and less efficient mobbing event (Andersson 1976). Additionally, individuals may also suffer from lower survival due to missing critical signals (Griesser 2013; Griesser and Suzuki 2017). Finally, disrupting mobbing calls may also decrease the indirect benefits to the signalers (eg increased niche exploitation, learning about predators, etc.; Sullivan 1985; Suzuki and Ueda 2013; Carlson and Griesser 2022; León et al. 2022).

Second, human-induced habitat turn-over via methods such as by logging or urbanization, species invasions, and hunting pressure may alter predator density and diversity (*Predator & environmental interactions*; Doherty et al. 2016; Benítez-López et al. 2017). Increased rates of predation often result in similar increases in attendance in winter flocks and mobbing, while decreased rates of predation result in the opposite (Thiollay 1999; Sandoval and Wilson 2012). Shifting predator populations, such as the introduction of novel predators, could result in a delay of mobbing responses due to a lack of recognition followed by an increase in mobbing behavior if a species community can learn to recognize predators through mobbing (Curio et al. 1978; Baker 2004).

Third, human-induced habitat turn-over, chemical pollution, and capture and release of pet animals may also alter local communities of prey species (Turner 2010; Bullock et al. 2018), which could impact the occurrence and efficiency of mobbing in similar ways to other *Prey & environmental interactions*. Shifts in species communities, through increases and decreases in different species (Wallach et al. 2010; Sorte et al. 2017), could either facilitate or supress mobbing respectively, while also potentially resulting in a breakdown of current communication networks, resulting in fewer, or less efficient, mobbing events.

Fourth, changes in aggression, related to urbanization, may also directly affect mobbing propensity (as seen in *Prey factors*). Urban populations of avian songbird species can exhibit higher levels of aggression compared with their rural counterparts (eg house sparrows (Davies and Sewall 2016), great tits (Hardman and Dalesman 2018) and common chiffchaffs, *Phylloscopus collybita*, (Wolfenden et al. 2019)). If urban populations become, on average, more aggressive, mobbing propensity is likely to change. Aggressive birds may be more willing to engage in aggressive behavior (Vrublevska et al. 2015) like mobbing, and/or may turn that aggression toward previously tolerated conspecifics or heterospecifics. This could result in increases in individual species mobbing, but lower recruitment and effectiveness of mobbing, potentially leading to a decreasing propensity over time.

Fifth, various changes in landscape openness, food resources, and average temperatures (*Environmental factors*) may alter the balance in predator-prey relationships and affect the propensity and efficiency of mobbing behavior (Thiollay 1999; Sandoval and Wilson 2012). Urbanization typically involves dramatic changes in vegetation density, type and appearance, for example buildings forming discrete visual and auditory boundaries in an otherwise relatively open and exposed landscape (Seress and Liker 2015), while buildings and roads increase impervious surfaces (Ewing et al. 2025). Food resources also change in urban areas, often with increased resources for some species (Seress and Liker 2015). This coupled with higher winter temperatures could result in less well-attended mixed-species groups (Thiollay 1999; Sandoval and Wilson 2012), ultimately decreasing mobbing propensity.

Finally, climate change and increased temperatures may impact mobbing behavior (Cordonnier et al. 2023). Great tits show both reduced aggressive mobbing response and lower calling rate in response to playback of mobbing calls on hot days compared with cooler ones (Cordonnier et al. 2023). If a common response to increased temperature, then climate change may result in decreased mobbing propensity across species and communities. Even if this response is isolated to only a few species like great tits, which are thought to serve as community informants (Carlson et al. 2020b), a decrease in their propensity to mob could result in lower heterospecific mobbing recruitment and efficacy, lowering the benefits to mobbing for participant species. Additionally, if these reductions in mobbing activity are a result of heat stress as is suggested (Cordonnier et al. 2023), then mobbing risk may also be greatly increased as heat stress can affect many behavioral (Pattinson and Smit 2017) and cognitive processes (Soravia et al. 2021; Blackburn et al. 2022) that could make assessing or evading relevant threats more difficult.

## Final thoughts

Mobbing is a widely occurring and important behavior that may affect entire species communities. It has attracted the attention and fascination of many scientists over the years and incorporates aspects of cooperation and communication making it a useful system to study some of the fundamental questions about animal behavior, perception, cognition, and decision making. Yet for all this, its ecology and the selection pressures that have given rise to this behavior are still relatively poorly understood. We believe that mobbing can be an ideal system to examine a variety of questions about animal behavior, including why, how, and in what circumstances individuals cooperate and communicate with conspecifics and heterospecifics, how individuals evaluate and make decisions about risk to self and kin, and how the habitats and life-histories of different species impact these behaviors. To ask these questions using mobbing, however, we must first understand the drivers for this behavior and how the ecology of predators, prey and the environment shape the behavior itself. By starting from a shared understanding of the factors that can impact mobbing, we can move forward to unveil the processes and conditions that lead to the evolution and current expression of mobbing behavior across different species and contexts.

## Data Availability

This work did not rely on collected experimental or observational data so no data is archived.
